# Tumour Microenvironment-Immune Cell Interactions Influencing Breast Cancer Heterogeneity and Disease Progression

**DOI:** 10.3389/fonc.2022.876451

**Published:** 2022-05-13

**Authors:** Keely Tan, Matthew J. Naylor

**Affiliations:** Charles Perkins Centre, School of Medical Sciences, Faculty of Medicine and Health, University of Sydney, Sydney, NSW, Australia

**Keywords:** breast cancer, tumour microenvironment, immune cell, stroma, metastasis, disease progression

## Abstract

Breast cancer is a complex, dynamic disease that acquires heterogeneity through various mechanisms, allowing cancer cells to proliferate, survive and metastasise. Heterogeneity is introduced early, through the accumulation of germline and somatic mutations which initiate cancer formation. Following initiation, heterogeneity is driven by the complex interaction between intrinsic cellular factors and the extrinsic tumour microenvironment (TME). The TME consists of tumour cells and the subsequently recruited immune cells, endothelial cells, fibroblasts, adipocytes and non-cellular components of the extracellular matrix. Current research demonstrates that stromal-immune cell interactions mediated by various TME components release environmental cues, in mechanical and chemical forms, to communicate with surrounding and distant cells. These interactions are critical in facilitating the metastatic process at both the primary and secondary site, as well as introducing greater intratumoral heterogeneity and disease complexity by exerting selective pressures on cancer cells. This can result in the adaptation of cells and a feedback loop to the cancer genome, which can promote therapeutic resistance. Thus, targeting TME and immune-stromal cell interactions has been suggested as a potential therapeutic avenue given that aspects of this process are somewhat conserved between breast cancer subtypes. This mini review will discuss emerging ideas on how the interaction of various aspects of the TME contribute to increased heterogeneity and disease progression, and the therapeutic potential of targeting the TME.

## Introduction

Breast cancer is a complex and heterogeneous disease that accounted for 24.5% of cancer diagnoses and 15.5% of cancer-related deaths in women in 2020 alone, making it the most commonly diagnosed and most lethal cancer in women worldwide (1). In Australia, the 5-year survival rate for those diagnosed with early-stage breast cancer is 91%, but this dramatically decreases to 32% in patients with invasive metastatic disease (2). Metastasis is a primary hallmark of cancer and is a multi-step process that begins with intravasation of cancer cells from the primary tumour site, migration and survival in the vascular or lymphatic systems, and is completed following extravasation and colonisation at a distal site (3, 4). The ability of cancer cells to survive, progress and metastasise is largely determined by intratumoral heterogeneity (ITH), which is dependent on both genetic and environmental influences (5). Whilst environmental influences encompasses both external and internal factors, this review will focus on the internal tumor microenvironment (TME).

**Figure 1:**
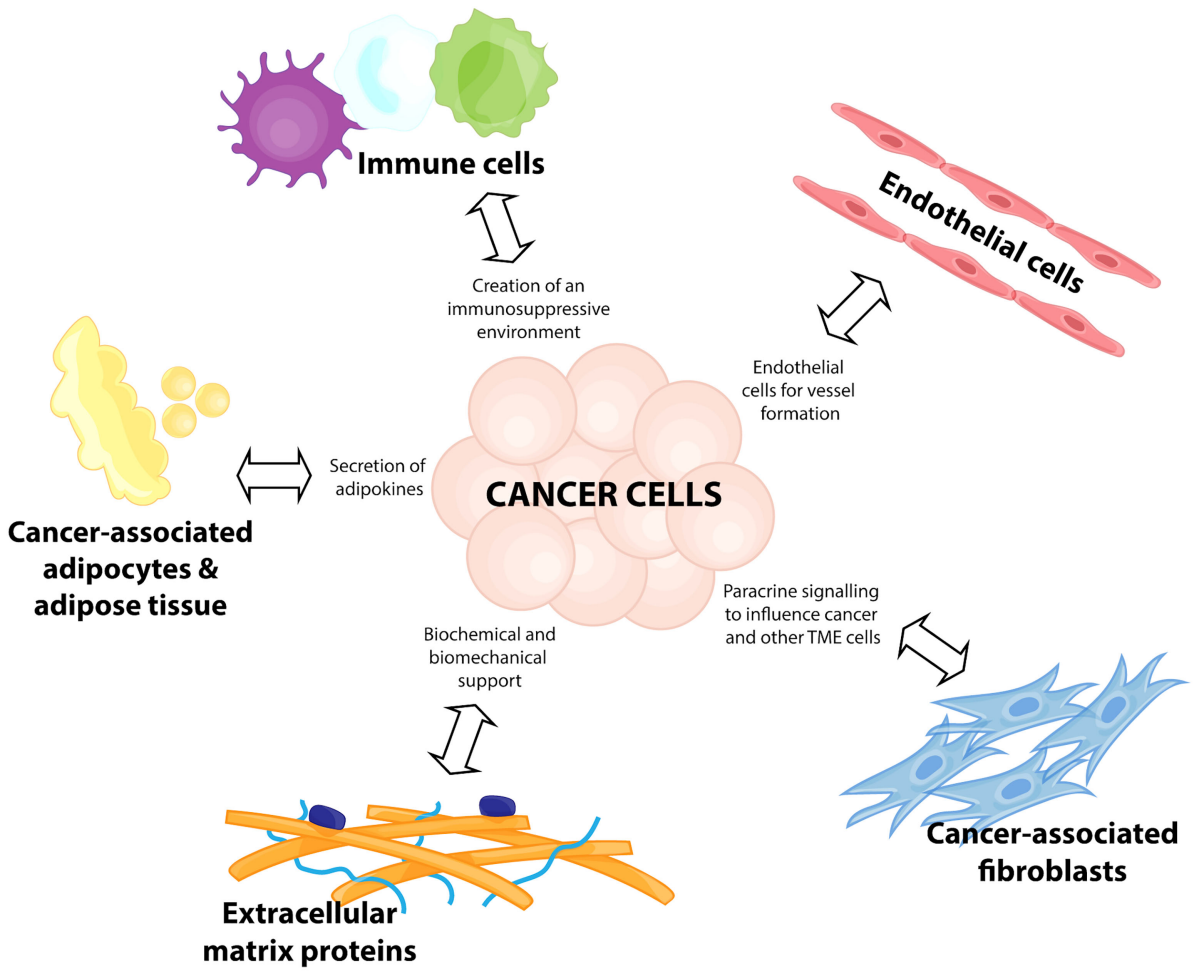
Components of a breast cancer tumor microenvironment. Breast cancer disease progression relies on a complex network of cells and interactions, termed the tumor microenvironment (TME). This consists of immune cells for the creation of an immunosuppressive environment, recruitment of endothelial cells for vessel formation, transformation of cancer-associated fibroblasts to participate in paracrine signalling to influence the cancer cells and other TME cells, release of adipokines from cancer-associated adipocytes and adipose tissue, and the biochemical and biomechanical support from extracellular matrix proteins.

The formation of the TME is initiated in response to metabolic needs of rapidly proliferating cancer cells. At the heart of this complex network are the tumor cells themselves, which manipulate surrounding cells through signaling networks activated by biochemical and biomechanical mechanisms. The cell types present in the TME include a variety of non-malignant stromal cells, such as endothelial cells, adipocytes, fibroblasts, immune cells, and extracellular matrix (ECM) proteins (6) (**Figure 1**). They influence cancer progression by contributing to cellular genomic and biological variations, increasing clonal evolution, and thus increasing ITH (7, 8). It is thought that increased ITH is a key feature of cancer cell survival and progression (6, 9). This mini review will discuss emerging ideas on how key players of the breast cancer TME influence ITH, metastasis and therapeutic resistance. Whilst the focus of this review will be on the tumor-promoting functions of the TME, it is important to acknowledge that these factors exist in a relatively plastic state, and thus are only pro-tumorigenic in optimal conditions (10).

## Tumor Microenvironment and Heterogeneity

ITH is defined as the genomic and biological variations acquired from exposure to various microenvironmental elements, contributing to the phenotypic characteristics of the cancer and increasing its survival capabilities (7, 8). Whilst it is known that intrinsic cell heterogeneity exists within a tumor cell population, greater heterogeneity can be introduced through environmental influence. Increasing the diversity of TME components in the peritumor space provides a plethora of signals to cancer cells, contributing to increased ITH through genetic and reversible epigenetic modulation (7, 10). For example, hypoxia has been linked to the regulation of epigenetic modulators, including G9a histone H3 lysine 9 (H3K9me) methyltransferase (11) and histone demethylase jumonji domain containing protein 2C (JMJD2C) (12). This increases the activity of hypoxia inducible transcription factor (HIF)-1, which contributes to breast cancer progression. Furthermore, cancer associated fibroblasts (CAFs) and their associated ECM products also trigger epigenetic modifications that influence cancer phenotype.

### Stromal-Immune Cell Interactions and TME Heterogeneity

Initially, tumor-secreted factors recruit and transform surrounding non-malignant cells into cancer-associated cells, but these cancer-associated cells also possess their own ability to influence other TME cell types through dynamic, bi-directional communication (6). Typically, a range of immune cells are involved in launching an anti-tumor immune response, including natural killer (NK) cells, CD8^+^ and CD4^+^ T cells and dendritic cells (DCs) (13, 14). However, these cells can be exploited by various TME elements to become pro-tumorigenic. As a tumor begins to expand, its growth is limited by the increasing hypoxic microenvironment and decreasing metabolic substrates, triggering an angiogenic switch *via* HIF-1 and -2 activation. This results in a range of physiological effects, including the production of pro-angiogenic growth factors, such as vascular endothelial growth factor (VEGF) (15). These factors encourage recruitment and proliferation of endothelial cells from pre-existing capillary beds, resulting in the formation of a disorganised and highly permeable tumor vascular network, allowing for the transport of more oxygen and nutrients (16). In addition, a VEGF gradient and interstitial flow from leaky tumor vasculature promotes lymphangiogenesis, resulting in lympathic endothelial cell migration in the direction of flow and gradient (17, 18). The presence of these leaky vessels and the mechanical stress caused by increasing ECM production by TME components, results in the transportation of antigen-presenting cells (APCs) from adjacent tissue. The presence of these antigens stimulates the recruitment of macrophages and DCs to the tumor site (19). Whilst normally anti-tumorigenic, secretion of factors by TME components, such as transforming growth factor (TGF)-ß by CAFs, has the potential to neutralize the anti-tumor response of NK cells, neutrophils and macrophages (20), enhancing immune evasion. Concurrently, the recruitment and enhancement of immunosuppressive cells, such as regulatory T cells (Tregs) and tumor-associated macrophages (TAMs), can create an immunosuppressive TME (21, 22).

Hypoxic breast cancer cells also secrete a variety of paracrine signaling molecules that can reprogram progenitor cells into CAFs (23). CAFs represent a major portion of the breast tumor stroma and are a highly heterogeneous population derived from various cell types, such as resident fibroblasts, bone marrow cells and adipocytes (24–26). CAF-secreted factors such as TGF-ß, act in an autocrine manner to further promote differentiation of fibroblasts into CAFs (27). These CAF-secreted factors can also influence the functions of other cell types within the TME (28). Secretion of stimulating factors, such as IL-6, results in ECM remodeling and matrix stiffening, providing a physical barrier to immune cells, particularly T lymphocytes (28), while conversely increasing the number of infiltrating TAMs (29). Whilst TME components can reduce infiltration of anti-tumorigenic immune cells, the presence of extracellular vesicles (EVs) has also been shown to decrease proliferation of CD8^+^ and CD4^+^ T cells (30). In addition to being a CAF precursor, cancer-associated adipocytes (CAAs) have been associated with breast cancer progression. The breast is rich in adipose tissue, and the interaction between the adipocytes and breast cancer cells is significant in disease progression (31). CAAs exhibit a unique morphology and phenotype, with smaller and dispersed lipid droplets, a decrease in volume with a dilated interstitial space, and alterations in shape (32, 33). These CAAs can be peritumoral or intratumoral and release various adipokines, such as IL-6 and leptin. Interestingly, the effect of CAAs differs depending on the location, with intratumorally located CAAs reflective of decreased cancer cell proliferation and improved patient survival (34), whilst peritumoral CAAs are associated with poor prognosis (33). However, both intra- and peritumoral CAAs are associated with increased inflammation and metastasis. It is postulated that the release of adipokines attracts immune cells, such as monocytes, to the primary tumor site, which facilitates immune evasion (35, 36). Ultimately, the recruitment and reprogramming of various non-malignant cell types creates a diverse TME, which functions to support and facilitate breast cancer progression.

## Contribution of TME to Breast Cancer Metastasis

**Figure 2:**
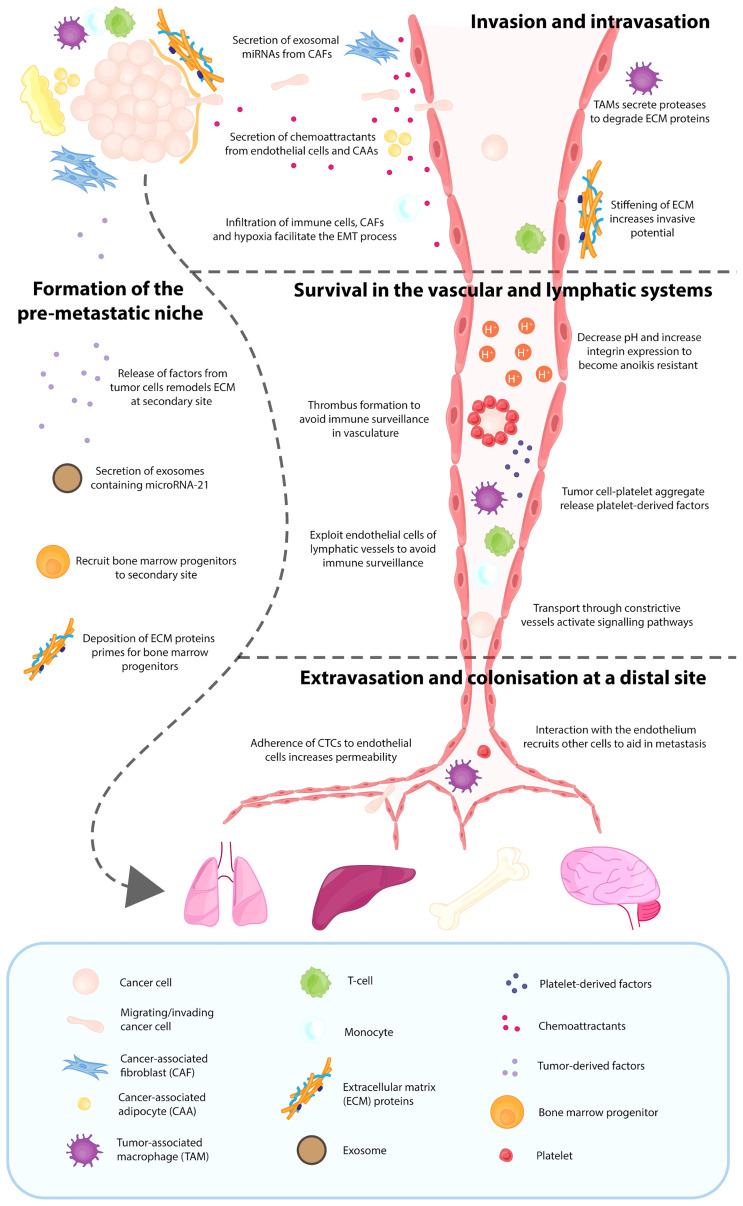
Contribution of various tumor microenvironment components in the process of metastasis. Breast cancer metastasis relies on various components of the tumor microenvironment, with the major sites of metastasis being the lungs, liver, brain and bone. Even before breast cancer cells escape from the primary site, several factors and exosomes have been released into the circulation by the breast cancer cells, which results in deposition of extracellular matrix (ECM) proteins at the distal site and recruitment of bone marrow progenitors. This creates a favourable pre-metastatic niche (PMN), which is crucial for metastatic dissemination. The TME and PMN support breast cancer cells in the process of invasion and intravasation, survival in the lymphatic and vascular systems, and the eventual extravasation and successful colonisation. Once these CTCs have reached the distal organ, the formation of a metastatic niche is initiated, which supports the growth of the secondary lesion.

Once the TME framework has been established to support the growth and survival of the primary breast tumor, these various components can promote the metastatic dissemination of cancer cells (37, 38). For breast cancer, the major sites of metastasis are lungs, liver, brain and bone (39), however prior to undergoing metastasis breast cancer cells release chemokines and cytokines into the circulation. The premetastatic release of these chemokine and cytokines creates a favourable premetastatic niche (PMN), which recruits circulating tumor cells (CTCs) to the secondary site (37, 40). Thus, it is the combination of both the primary TME and the PMN that drives metastasis. These two microenvironments support breast cancer cells in the process of invasion and intravasation, survival in the lymphatic and vascular systems, extravasation and successful colonisation. Once CTCs have reached the distal site, the formation of a metastatic niche is initiated, which supports the growth of the secondary lesion (41) (**Figure 2**).

### Formation of the Premetastatic Niche

The formation of the PMN is initiated with local changes and followed by systemic changes. Initially, vascular leakiness occurs because of the disorganised tumor-derived vasculature, resulting in alterations to resident cells, such as fibroblasts, and the recruitment of non-resident cells, such as bone marrow progenitors (40). In breast cancer, HIF signalling has been demonstrated to result in the secretion of lysyl oxidase (LOX), LOX-like proteins (LOXL2 and 4) and exosomes, which has previously been shown to be crucial in establishing a premetastatic niche in the lungs and bones (42). In contrast, *Hif1α* deletion in a PyMT mammary cancer model reduced bone metastasis but was associated with increased pulmonary metastasis (43). Previous research has also correlated a hypoxia transcriptome with both bone (44) and lung (45) metastasis signatures, suggesting that although there are genetic changes reflected in the transcriptome, there may also be distinctive signals within the TME that cells respond to in order to determine the phenotypic response (43). The release of these factors by the primary tumor can result in ECM remodeling at the secondary metastatic site, priming the location for bone marrow progenitor cell colonisation. VEGFR1^+^ haematopoietic bone marrow progenitors, which are mobilised during the tumor’s angiogenic switch, have also been demonstrated to be crucial in the initiation of the PMN. The homing of these VEGFR1^+^ bone marrow progenitors to the premetastatic site is dependent on the prior increased deposition of ECM proteins, such as fibronectin, which binds to its receptor integrin α_4_ß_1_ on progenitor cells (40). More recently, the contribution of exosomes released from breast cancer cells has been demonstrated to be critical in the formation of the premetastatic niche for bone metastasis. This was through the activity of the exosomal derived microRNA-21, which is involved in the process of bone remodeling (46), which may suggest that site specific adaptations determine the organ of breast cancer metastasis.

### Invasion and Intravasation

Whilst the formation of this secondary TME is critical for the deposition of CTCs, the cells must first escape from the primary site. To successfully intravasate, cancer cells rely on the contribution of intrinsic cell properties, microenvironmental factors and mechanical cues (47). A fundamental aspect of metastasis is the ability of cancer cells to undergo EMT, and this process is facilitated by various elements of the TME, such as the presence of hypoxia, infiltration of immune cells and activity of CAFs (43, 48). These TME factors are also able to further promote the migratory and invasive ability of these transformed cancer cells. Cancer cell migration has been demonstrated to be supported by the expression of focal adhesion kinase (FAK) in CAFs through the secretion of exosomal miRNAs (49). In addition, various TME components, such as CAAs and endothelial cells, can secrete chemoattractants to promote the movement of cancer cells in the direction of the gradient (17, 36). For example, a study by Cho and colleagues used a 3D macrofluidic device to demonstrate the communication between lymphatic vessels and tumor cells through the CXCL21/CXCR7 axis, with lymphatic endothelial cells secreting CXCL21, a chemoattract for breast cancer cells expressing CXCR7, causing invasion of MDA-MB-231 towards the lymphatic vessels (17). The secretion of degradative enzymes by TAMs and EVs, function to lyse surrounding ECM, further supporting the process of invasion (50, 51). These microenvironmental factors also have the potential to modify ECM proteins, which results in ECM stiffening. The stiffening of the ECM sends mechanical cues to further perpetuate the activation of CAFs in a positive feedback loop, and this tissue rigidity is associated with more aggressive breast cancers, with worse patient prognosis (52). Rigidity has been associated with upregulation of Mammalian-enabled (Mena), particularly the Mena^INV^ isoform, which is a protein that has been shown to be upregulated in invasive breast cancer cells and associated with invadopodia maturation (52, 53). Furthermore, the direct contact between an Mena^INV^ overexpressing breast cancer cell, an endothelial cell and a Tie2^high^/VEGF^high^ perivascular macrophage is crucial in forming the TME of metastasis (TMEM), a process required for intravasation (54).

### Survival in the Vascular and Lymphatic Systems

Whilst the newly formed vascular and lymphatic networks can act as a transport route for cancer cells to secondary distal sites, travelling through these harsh environments exerts selective pressures that cancer cells must overcome (55). Upon exiting the primary tumor site, cancer cells experience anoikis, a form of cell death initiated from the detachment of cells from the ECM (56). In order to overcome this and become anoikis resistant, cancer cells can employ various mechanisms, such as reprograming the metabolism of the TME, decreasing the pH (57) and upregulating the expression of specific integrins (58). Once tumor cells enter the vascular system, they activate platelets to form a thrombus around the cells, protecting cancer cells from immune surveillance (59, 60). It is thought that this tumor cell platelet aggregate releases a plethora of platelet-derived factors, such as TGF-ß1, which has the potential to decrease the antitumor activity of NK cells (61). Tumor cells that disseminate through the lymphatic system initially experience a hypoxic microenvironment, which is overcome by exploiting the native vasculature of the lymph node (62). To survive in the lymphatic system, cancer cells must also evade immune surveillance, which can be achieved through exploitation of the lymphatic endothelial cell’s ability to scavenge and cross-present lymph antigens, resulting in removal of autoreactive naive CD8^+^ T cells (62). Furthermore, the migration of these CTCs through highly constrictive vessels can *via* mechanotransduction mechanisms, result in the activation of various signalling pathways which may contribute to increased cancer cell survival, heterogeneity and motility (63, 64).

### Extravasation and Colonisation at Distal Site

The process of metastasis is inefficient, with <0.01% of cells disseminated from the primary tumor successfully colonising at a distal site. This process generally occurs within the capillaries, where they are able to lodge and interact with the endothelium (55). This interaction with the endothelium can promote the recruitment of various other cells, such as macrophages, platelets and neutrophils which aid in the successful metastasis of cancer cells (65, 66). Furthermore, components of the TME, such as hypoxia-associated HIF activity of cancer cells, have been implicated in the process of extravasation. This involves stimulating the release of factors such as L1 cell adhesion molecule and angiopoietin-like 4 that promote the adherence of CTCs to the endothelium of lungs and interferes with endothelial cell adhesion molecules, respectively, increasing vascular permeability (67). It is important to note that the optimum metastatic conditions are tissue specific, an important consideration when attempting to identify new treatment options for metastatic disease. For example, CTCs honing towards the brain secrete factors, such as α-crystallin, neuroserpin and cathepsin S (68), whereas CTCs with an affinity for the lungs tend to depend on myeloid cell populations (69). During the initial formation of any metastatic foci, the cancer cells remain close to existing vasculature before stimulating angiogenesis for its own blood supply (68). Once the cells have successfully colonised, the microenvironment is again altered by the newly deposited tumor cells, and this is then termed the metastatic niche (41).

### Therapeutic Resistance

In addition to its contribution to breast cancer progression and metastasis, various components of the TME have been implicated in the therapeutic resistance of breast tumors (70, 71). Brechbuhl and colleagues identified that the contribution of CAF subpopulations to therapeutic resistance varies, with CD146^+^ CAFs in estrogen receptor (ER) positive breast cancer cells displaying sensitivity to tamoxifen treatment, while CD146^-^ CAFs were correlated with decreased ER expression in the same cells and thus increased resistance to tamoxifen treatment (71). A recent study has identified that there is a novel relationship between ductal carcinoma *in situ* and fibroblasts that express platelet-derived growth factor receptor (PDGFR)α^(low)^/PDGFRß^(high)^ through elevated Notch signalling in fibroblasts (72). This may be due to the paracrine interactions of PDGF-CC released from breast cancer cells interacting with fibroblasts through PDGFRs, orchestrating an ERα-negative phenotype through stimulation of hepatocyte growth factor (HGF), insulin-like growth factor binding protein (IGFBP) 3 and stanniocalcin (STC) 1 secretion from CAFs (73). Subsequent treatment of PeRo-Lum1 cells, a luminal mammary cancer cell line, with CAF-conditioned medium resulted in decreased cell sensitivity to tamoxifen through the decrease in ERα expression, suggesting that the interactions between CAFs and cancer cells can alter the molecular subtype of breast cancers and ultimately, endocrine therapy resistance. Furthermore, exosomes can promote therapeutic resistance by enabling interaction between TME components as well as through direct interaction with breast cancer cells (70, 74). Given the major contribution of TME components to breast cancer progression, some TME targeting therapeutics have been identified.

### Targeting the TME for Breast Cancer Treatment

Breast cancer was previously viewed as a disease with low immunogenicity, however recent research demonstrates the possibility of immunotherapy in the treatment of breast cancer (75). This is further supported with atezolizumab and pembrolizumab being recently FDA-approved for use in combination with chemotherapy for programmed death ligand 1-positive triple negative breast cancers (76). In addition, the most recent WHO histopathological assessment of breast tumor guide now includes investigation of tumor-infiltrating leukocytes and fibrotic foci (77), highlighting the increasing recognition of the TME in determining disease severity. A recent study by Harney and colleagues also demonstrated the effects of a potent Tie2 inhibitor, rebastinib, on a mammary cancer model (78). Independent treatment resulted in reduced Tie2+ macrophages, TMEM function and angiogenesis, which presented as decreased mammary tumor growth, metastasis and overall increased survival, however it was the combination of rebastinib and paclitaxel that was most effective. Most of the available studies highlight the success in combining therapies, whether that be traditional therapies such as radiotherapy and chemotherapy, with a TME-targeting agent, such as anti-angiogenics and immunotherapy (76, 78) or two TME-targeting agents (79). Whilst these studies are a step in the right direction, there is still much unknown with respect to translating TME targets into the clinic and thus this should be a focus of breast cancer research, particularly metastatic breast cancer.

## Concluding Remarks

It is only within the past 3 decades that cancer research has shifted from primarily focusing on cancer cells to appreciating that cancer is a complex system with many biological aspects that contribute to tumor growth and progression (80). Without the structural integrity of the TME, breast cancer cells would not successfully grow and metastasise. It is factors released from the primary TME that establish a premetastatic niche at a distal secondary site, thus targeting aspects of the TME has the potential to decrease the prevalence of metastasis, the greatest contributor to breast cancer mortality rates. In addition, the components of the TME are somewhat conserved between breast cancer subtypes, offering a broad-spectrum therapeutic target for breast cancer.

## Author Contributions

KT designed the study, was responsible for writing the article and the creation of all figures. MN designed the study and was responsible for writing and revising the manuscript. All authors contributed to the generation of the concepts and ideas provided.

## Funding

This work was supported by Cancer Council NSW Research Project Grant (RG 20-08) and Priority-driven Collaborative Cancer Research Scheme (Grant #1130499), funded by the National Breast Cancer Foundation Australia with the assistance of Cancer Australia awarded to MN.

## Conflict of Interest

The authors declare that the research was conducted in the absence of any commercial or financial relationships that could be construed as a potential conflict of interest.

## Publisher’s Note

All claims expressed in this article are solely those of the authors and do not necessarily represent those of their affiliated organizations, or those of the publisher, the editors and the reviewers. Any product that may be evaluated in this article, or claim that may be made by its manufacturer, is not guaranteed or endorsed by the publisher.
